# The Extent of Psychosocial Distress among Immigrant and Non-Immigrant Homecare Nurses—A Comparative cross Sectional Survey

**DOI:** 10.3390/ijerph17051635

**Published:** 2020-03-03

**Authors:** Benjamin Schilgen, Albert Nienhaus, Mike Mösko

**Affiliations:** 1 Department of Medical Psychology, University Medical Center Hamburg-Eppendorf, Martinistraße 52, 20246 Hamburg, Germany; m.moesko@uke.de; 2 Competence Centre for Epidemiology and Health Services Research for Healthcare Professionals (CVcare), Institute for Health Services Research in Dermatology and Nursing, University Medical Centre Hamburg-Eppendorf, Bethanien-Höfe Eppendorf, Martinistraße 52, 20246 Hamburg, Germany; albert.nienhaus@bgw-online.de; 3 Department of Occupational Health Research, German Social Accident Insurance Institution for the Health and Welfare Services, Pappelallee 33/35/37, 22089 Hamburg, Germany

**Keywords:** foreign professional personnel, home health nursing, occupational health, comparative study

## Abstract

In times of demographic change, most developed countries are increasingly looking to cover the growing domestic demand for healthcare by hiring nurses from abroad. The evidence concerning the health of immigrant care workers is inconsistent since studies report that it is either better or more impaired than that of their non-immigrant counterparts. This study compared the extent of occupational psychosocial stressors and resources affecting immigrant and non-immigrant homecare nurses. The cross-sectional survey was conducted in the homecare nursing service sector in Hamburg. Psychosocial distress, depressive symptomatology, generalized anxiety, somatic symptom burdens, homecare-specific qualitative stressors, as well as resources, have been measured using a standardized questionnaire. There was no significant difference in the extent of psychosocial distress experienced by immigrant and non-immigrant homecare nurses. Somatic symptom burdens most strongly predicted nurses’ psychosocial distress, in general. For immigrant nurses, greater influence and freedom at work, as well as fixed-term employment, was related to increased levels of distress, while age, working full time, and working overtime predicted distress in non-immigrant nurses. A functioning relationship with colleagues and superiors had a declining effect on immigrant nurses’ psychosocial distress, while shift work arrangements benefitted non-immigrant nurses. Even though the extent of psychosocial distress experienced by immigrant and non-immigrant nurses did not significantly differ, the nurse’s individual explanatory model of psychosocial health should be considered in every occupational and political context.

## 1. Introduction

Providing adequate healthcare to people is a global challenge [1]. A global shortage of healthcare workers is being experienced alongside demographic change, the increase of chronic diseases, as well as migration [2].

The global “Nursing Now” campaign aims to create awareness about the need for more nurses, but also the development of new and innovative types of services in times of a more home-based, people-focused, and more preventive healthcare [3]. According to the World Health Organization, nurses and midwives account for 50% of the current global shortage of health workers, which is forecasted to reach 9 million by 2030 [4]. In 2018, 1.6 million nurses were employed in Germany [5], while calculation scenarios assume a shortage of 260,000 to 430,000 full-time equivalents by 2030 [6]. This is due to an increase of people in need of long-term care concomitant with declining labor resources. Following the European trend of “Aging in place” [7,8,9,10] and, therefore, financially strengthening family care, Germany fosters the priorit**y** of outpatient care. This should reduce the increasing demand for nursing care that is carried out by professional nurses [11].

Immigration intensifies the cultural and linguistic diversity, challenging the German healthcare system. The proportion of people with an immigrant background in Germany will rise from 24% in 2017 [12] to 28% in 2030 [13]. The Federal Ministry of Health estimated that 310,000 immigrants were in need of care in 2018, and predicted an increase to 474,000 in 2030 [13].

The profession of nurse is associated with frequent mental and physical stress, having a negative impact on the nurses’ health. This may, in turn, cause nurses to vacate their jobs [14,15,16]. The mobili**z**ation of patients or clients in terms, for example, body care (washing the patient’s/client’s body) and time pressure, caused by the rising work intensification, are experienced as particularly stressful. This is followed by administrative tasks, such as care documentation and repeated interruptions of the workflow [17,18]. Homecare nurses are also exposed to specific musculoskeletal stresses, such as moving furniture, to optimi**z**e their workplace [19].

Immigrant and minority homecare staff additionally report discrimination and racism at work in terms of a lack of employment opportunities, poor career progression, or poor learning environment [20]. They experience racial discrimination during their contact with patients and their families, but also with doctors, management, and nursing colleagues [21]. Discrimination and racial harassment have a significant negative impact on job satisfaction [22].

Nurses partially compensate their occupational burdens through the inherent diversity of the nursing job, the great scope for action, teamwork, and a strong personal affirmation through their job. A positive and functioning relationship with colleagues and superiors within the institution are further resources [17,18].

Nurses share similar work-related barriers regardless of their ethnic or cultural background. The leading stressor, which hinders a functioning collaboration between nurses, is communication problems due to language barriers with their non-immigrant counterparts. Within a diverse workforce, a different understanding and diverse expectations of nursing quality inside the team can cause conflicts [20].

Studies show that immigrant healthcare workers are in poorer health in the host country than their non-immigrant counterparts and explain this with the often adverse living conditions, such as lower socioeconomic status, being employed in a more hazardous occupation, or affected by unemployment or a disadvantageous housing situation [23]. The German Aging Survey (DEAS) has shown that 29% of immigrants in the second half of their lives in Germany are affected by functional health restrictions compared to 24.4% of non-immigrant workers. Likewise, more than 33% of immigrant workers report mild forms of depressive symptoms compared to 26% of non-immigrant workers, respectively. While immigrants in the second half of their lives in Germany manifest a lower health status compared to non-immigrants, data prove that the living and health status of second-generation immigrants are similar to their non-immigrant counterparts [24]. The “German Health Interview and Examination Survey for Adults” (DEGS) concludes that depressive symptoms are more frequent among male immigrants (10.6%) than non-immigrant males (5%), as well as among female immigrants (15.1% versus 9.1%) compared to female non-immigrants [25]. Despite the lower health status of immigrants, in general, study results show a similar and better health status of second-generation immigrants born in Germany and having at least one parent born abroad [23,24,25].

Summing up, one can say that the data about the health of immigrant healthcare workers in Germany are heterogeneous, comparison data of immigrant and non-immigrant nurses are lacking, while the number of immigrant nurses is expected to rise, which will lead to the further diversification of the healthcare workforce in a number of countries, including Germany.

**Figure 1:**
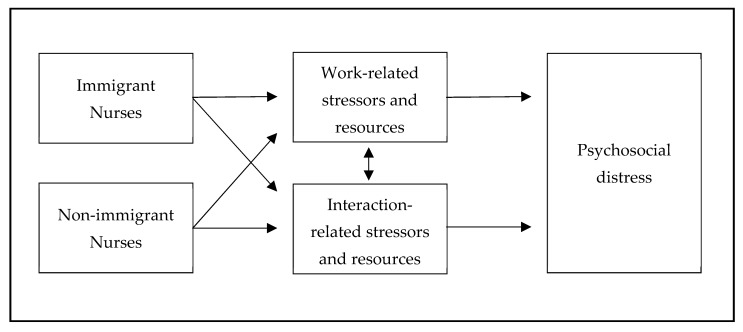
A theoretical framework for the study.

Based on the transactional model of stress by Lazarus and Folkman (1984) and its adaption to the nursing context, we juxtaposed – for this study’s purpose – immigrant and non-immigrant nurses. Those then are interacting as individual human beings in the work context of homecare nursing. Psychosocial stress emerges, on the one hand, by person-related (e.g., illness) and situation-related stressors (e.g., time pressure), as well as person-related resources (e.g., self-efficacy) and situation-related resources (e.g., team/supervisor support) on the other hand [26,27,28]. Immigrant and non-immigrant homecare nurses share similar stressors in the context of work (e.g., lifting heavy patients, time pressure) and interaction (e.g., unequal treatment) and resources (mutual support, exchanges with colleagues) to master occupational burdens. However, they differ in their explanations on how they perceive those stressors and in their behavior on how they cope with them. This leads to the interdependence of work-related and interaction-related stressors and resources, which, in turn, can affect the collaboration within a cultural and linguistic diverse nursing team, as well as towards superiors and in the intercultural nurse-client relationship [20,21]. The following chart displays the conceptual framework of this study (Figure 1).

There is evidence that immigrant workers are in poorer health than their native counterparts are. The home care sector is a market with increasing demand and increasing linguistic and cultural diversification on the care provider and care recipient side. Studies about the health of migrant care workers, especially those about psychosocial health, are scarce. This study aimed to compare the extent of psychosocial stress experienced by immigrant and non-immigrant homecare nurses. The above-mentioned qualitative differences in the patterns of origin of psychosocial stress had also been quantified in this study to detect possible differences among immigrant and non-immigrant homecare nurses.

High turnover rates in times of lacking workforce resources and an increasing diversification is a serious threat for the provision of nursing care. Since workplace retention is positively correlated with the health of nurses, providing them with a healthy workplace will increase the retention rate [29,30].

The corresponding research questions were:

1. Are immigrant homecare nurses more stressed at work than their non-immigrant colleagues?
2. Which factors predict the psychosocial distress of those nurses?

## 2. Materials and Methods

### 2.1. Design

A cross-sectional design was applied.

### 2.2. Setting and Participants

Data were collected from nurses working in the homecare sector in the federal state of Hamburg in Germany. In 2017, more than 35% of Hamburg’s 1.8 million inhabitants had a migration background, and half of those were German citizens [31]. Hamburg was chosen due to the already existing regional access to the field within the study’s underlying research project [20,21,32]. A total of 11,200 nurses work in the homecare sector in Hamburg—more than two thirds on a part-time basis [33]. In Germany, 1.6 million nurses work in hospitals, in-home healthcare, and nursing homes 226,000 of these nurses are immigrants [13,34]. A statistical power analysis was performed with G*Power 3.0.10 a priori to estimate the sample size. We aimed to compare the extent of psychosocial distress among immigrant and non-immigrant homecare nurses. The primary outcome was the German version of the NCCN Distress Thermometer [35,36]. Based on Cohen’s criteria, a small to medium effect size of d = 0.35 was set in this study. The error was set to alpha = 0.05, the power to 1-ß = 0.8, as well the allocation ratio to 1 due to the comparison between the two groups of nurses. Thus, our proposed sample size was 260; in other words, 130 immigrant nurses and 130 non-immigrant nurses. Based on recent similar studies [37], we estimated response of 30% and so had to distribute at least 870 questionnaires. After mapping every homecare provider in Hamburg to its specific district, the strata were then sorted according to the proportion of people with a migration background in each district. Within those, we randomly selected between four to 17 nursing services and contacted their management by telephone, inviting them to join the survey. The main author also visited some of the services to present the study. Once consent was given, the nursing services’ directors were asked to deliver questionnaire packs that also contained all relevant data privacy explanations. Completed questionnaires were either returned in an anonymous envelope or were personally collected by the main author. Participants’ consent in the study was given by the return of the questionnaire.

### 2.3. Sample

Due to the different proportion of people with a migration background in each district, the number of delivered questionnaires per district ranged from 89 to 262. After having distributed 870 questionnaires, we received 249 (immigrant nurses: 72, non-immigrant nurses: 177). After discussions within the project team, the proportional randomized stratified sampling approach was continued. Finally, 1110 questionnaires were distributed in 59 nursing services. In total, we received 287 questionnaires (n = 105 immigrant nurses, as well as n = 182 non-immigrant nurses), which corresponded to an overall response rate of 25.4%. The lower response rate among immigrant nurses corresponded with previous related research in this context [38].

### 2.4. Data Collection

Data were collected from 13 March 2018 until 1 February 2019.

### 2.5. Measures

#### 2.5.1. Primary Objective

The German version of the NCCN Distress Thermometer [35] was applied to assess psychosocial distress. The original version of the NCCN has been developed by the National Comprehensive Cancer Network (NCCN) in the U.S. to determine the type and extent of existing stress in oncological patients [36]. It consists of a one-item global screener for distress (the Distress Thermometer) and an accompanying problem list. The one-item screener is an 11-point Likert scale ranging from 0 (No distress) to 10 (Extreme distress). Patients indicate their levels of distress over the course of one week before assessment. The accompanying problem list consists of 40 items that detail practical (e.g., childcare, housing, insurance/financial), family (e.g., dealing with children/ with partner, ability to have children), emotional (e.g., fears, loss of interest in usual activities), spiritual (e.g., loss of faith in God), and physical problems (e.g., appearance, bathing/dressing, getting around, sexual, substance use) causing distress. The NCCN problem list is meant for patients to inform their doctor if they are having emotional concerns in the above-mentioned areas. For this study, only the scores from the one-item screener were analyzed since possible emotional concerns causing stress among immigrant and non-immigrant nurses have been qualitatively assessed and discussed elsewhere from this study’s authors [20,21]. With a sensitivity of 97% and a specificity of 41%, the NCCN shows adequate total psychometric properties. Following international recommendations, a person who scores 5 and above is moderately distressed and needs support. The tool is easy to use since it measures distress in a similar way to pain—on a scale of zero to 10 with 10 being the worst [35,36].

#### 2.5.2. Secondary Objectives

Somatic burdens of nurses were assessed using the German version of the Somatic Symptom Scale–8 (SSS-8) [39]. The SSS-8 measures results with a 5-point response option for stomach or bowel problems; back, arm, joints or legs pain; headaches; chest pain or shortness of breath; dizziness; feeling tired or having low energy, and, finally, having trouble sleeping. The sum score can range between 0 to 32 within a pragmatic categorization of none to minimal (0–3), low (4–7), medium (8-–11), high (12–15), and very high (16–32) [39]. Within this study, we applied a binary cut off with >11 for high symptomatic burdens.

Depression was assessed using the Patient Health Questionnaire-9 (PHQ-9) [40]. The PHQ-9 is the depression module of the PRIME-MD (*Prim*ary Care *E*valuation of *M*ental *D*isorders) diagnostic instrument for common mental disorders and scores each of the 9 DSM-IV (*D*iagnostic and *S*tatistical *M*anual of Mental Disorders) criteria from 0 (not at all) to 3 (nearly every day). The sum score (range 0 to 27) indicates the degree of depression with scores of ≥5, ≥10, and ≥15, representing mild, moderate, and severe levels of depression, respectively [40]. A score >10 defines the presence of depressive symptomatology [41]. Its sensitivity and specificity reaches 80% and 92%, respectively, [42] with a Cronbach’s alpha of 0.89 [40], and so the PHQ-9 shows good psychometric properties. In this study, the German version was applied [41].

Generalized anxiety was assessed with the Generalized Anxiety Disorder-7 (GAD-7), which measures anxiety symptoms for the two weeks prior to the time of the survey. Based on seven items on a Likert scale from 0 (not at all) to 3 (nearly every day), it can be used to determine the risk of GAD based on the DSM-IV criteria. The sum scale ranges from 0 to 21, and a higher total score reflects more severe symptoms of generalized anxiety disorder. A score of 10 and above is equivalent to a clinical diagnosis of GAD. A sensitivity of 0.89 with a specificity of 0.82 [43] and an internal consistency in the German population of alpha = 0.89 [44] prove good psychometric properties.

Qualitative stressors that are specific to the home care nursing context were measured against the BGW-miab (*B*erufsgenossenschaft für *G*esundheitsdienst und *W*ohlfahrtspflege-*Mi*t*a*rbeiter*b*efragung. Those are: taking care of the same patients on a daily basis, the confrontation with suffering and death of patients, and caring for patients with dementia or other psychiatric illnesses. Response options range from 0 (“Not at all”) to 4 (“Yes, exactly”) [45]. The internal consistency is robust, with a Cronbach’s alpha = 0.75. For further analysis, each strain item is dichotomized in “existing” and “non-existing” [46].

The Copenhagen Psychosocial Questionnaire (COPSOQ) measures psychosocial factors and work strain across all occupational sectors. The German version of the COPSOQ contains 85 items comprised of 26 scales [47]. Since the COPSOQ’s scales can be queried individually in questionnaires, only three scales of interest were used: influence and freedom at work, relationships with your colleagues and your superiors, as well as one question whether one’s work is recognized and appreciated by the management [48]. The instrument shows robust scale reliability with Cronbach’s alpha between 0.58 and 0.92 [49]. All items are answered on a five-point Likert scale, each added by the item “I have no superior/no colleagues” that is treated as a missing value once ticked.

Discrimination at work was assessed with an adapted version of the “ALLBUS—*All*gemeine *B*evölkerungs*u*mfrage der *S*ozialwissenschaften” survey [50]. This German General Social Survey collects recent data about attitudes, behavior, and social structure of the population in Germany. For the purpose of this study, nurses were asked about, for example, occupational discrimination in terms of payment, career progression, or application, as well as the possible reasons; the answer option was “yes” or “no”.

The collection of sociodemographic data (e.g., age, sex, marital status, children) was guided by the recommendations for epidemiological studies [51], as well as the study on Adult Health in Germany (DEGS) [52]. To assess the migration background, we applied the minimum indicator set for recording migration status [53]. Additionally, professional and educational level, income status, belonging to a religious community, and use and command of foreign languages were collated.

### 2.6. Analysis

**Figure 2:**
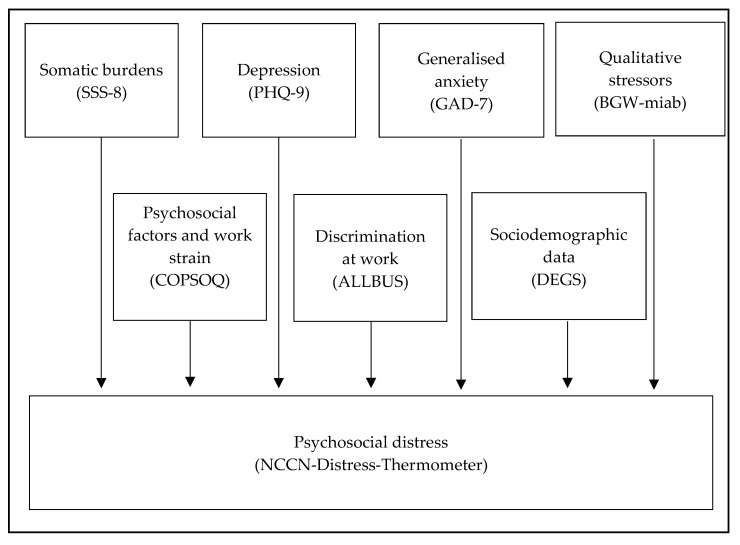
Flow chart of analysis. SSS-8: Somatic Symptom Scale, PHQ-9: Patient Health Questionnaire, GAD-7: Generalized Anxiety Disorder, BGW-miab: Berufsgenossenschaft für Gesundheitsdienst und Wohlfahrtspflege – Mitarbeiterbefragung, COPSOQ: Copenhagen Psychosocial Questionnaire, ALLBUS: Allgemeine Bevölkerungsumfrage der Sozialwissenschaften, DEGS: Study on Adult Health in Germany (Studie zur Gesundheit Erwachsener in Deutschland), NCCN: National Comprehensive Cancer Network Distress Thermometer.

The sample was divided into two groups: immigrant nurses and non-immigrant nurses. T-test was applied to analyze differences in interval scale variables and Chi-square tests to test differences in categorical scale variables. Since the latter test was applied repeatedly, the Bonferroni test was used for statistical significance. Hierarchical regression analyses were stepwise performed for each group to identify a possible impact of the above-mentioned secondary objectives affecting psychosocial distress as the primary objective (Figure 2). Missing data were handled with the expectation-maximization algorithm. Multicollinearity was not suspected, with all variance inflation factors (VIFs) being <2. Data analysis was carried out with SPSS 22.

## 3. Results

### 3.1. Sample Characteristics

More immigrant nurses were female compared to non-immigrant nurses, χ² (1) = 5.68, *p* = 0.017 (Table 1). Immigrant nurses were significantly younger than non-immigrant nurses, t (285) = −3.09, *p* = 0.002. Professional status differed significantly among both groups in terms of a higher proportion of registered general non-immigrant nurses versus a higher amount of lower qualified immigrant nursing assistants and trained nurses, χ² (4) = 16.22, *p* = 0.003. Significantly more immigrant nurses were married, while more non-immigrant nurses were divorced, χ² (5) = 16.22, *p* = 0.006. Non-immigrant nurses were employed in non-commercial operated services significantly more often, χ² (2) = 9.85, *p* = 0.007, while more immigrant nurses could not name what kind of service operator they work for. There was no significant difference between immigrant and non-immigrant nurses regarding weekly working time. There was a significant difference between both groups in terms of a higher proportion of immigrant nurses with fixed-term contracts, χ² (1) = 3.97, *p* = 0.046. Concerning German as a first language, the difference was significant within both groups, χ² (3) = 201.04, *p* = 0.

### 3.2. Basic Characteristics of the Primary and Secondary Objectives 

Immigrant nurses perceived psychosocial distress to a higher (M = 5.65, SD = 2.17) but not significantly different extent than non-immigrant nurses (M = 5.57, SD = 2.45), with t (285) = 0.284, *p* = 0.777. (Table 2). There was no significant difference between immigrant (M = 8.43, SD = 4.76) and non-immigrant nurses (M = 8.20, SD = 4.87) regarding somatic symptom burden, t (285) = 0.388, *p* = 0.698. Concerning depressive symptomatology, immigrant nurses showed higher values (M = 6.86, SD = 5.31) compared to their non-immigrant counterparts (M = 6.67, SD = 5.35), while they did not significantly differ, t (285) = 0.295, *p* = 0.771.

There was no statistical difference concerning general anxiety between both groups, t (285) = 0.713, *p* = 0.477, even though immigrant nurses scored higher M = 5.83, SD = 4.80 compared to M = 5.41, SD = 4.66. Concerning influence and freedom at work, immigrant (M = 49.95, SD = 27.70) and non-immigrant nurses (M = 51.14, SD = 21.81) did not differ significantly, t (179) = −0.376, *p* = 0.707. Immigrant nurses scored higher on the item “relationships with colleagues and superior” (M = 67.53, SD = 25.03) than their colleagues (M = 62.76, SD = 24.04), but also significantly different, t (285) = 1.596, *p* = 0.111. Immigrant nurses were more likely to feel appreciated by the management (M = 64.67, SD = 27.88) than their non-immigrant colleagues (M = 57.34; SD = 30.55) with t (285) = 2.021, *p* = 0.044.

### 3.3. Distribution of Groups according to Cut-Off Criteria of the Primary and the Main Secondary Outcomes

At 52.4% and 52.7%, respectively, there was no significant difference regarding the number of immigrant and non-immigrant nurses under noticeable psychosocial distress, χ² (1) = 0.004, *p* = 0.952 (Table 3). A total of 27.6% of immigrant nurses reported a high symptomatic burden compared to 24.7% of their non-immigrant counterparts, while there was no significant difference between both groups χ² (1) = 0.291, *p* = 0.589.

A total of 21.9% of immigrant nurses and 21.4% of non-immigrant nurses showed a depressive symptomatology without a significant difference χ² (1) = 0.009, *p* = 0.925.

A total of 18.1% of immigrant nurses reported generalized anxiety compared to 15.4% of their non-immigrant colleagues but did not differ significantly from each other χ² (1) = 0.357, *p* = 0.550.

The 32.4% of immigrant and 31.3% of non-immigrant nurses reported having been discriminated at work within the last 24 months without a significant difference χ² (1) = 0.035, *p* = 0.852. Nearly 60% of the immigrant and non-immigrant nurses who reported discrimination felt discriminated in the context of payment, 21% concerning career advancement, and nearly 65% with respect to the distribution of responsibilities. A total of 77.8% of the immigrant nurses and 72% of the non-immigrant nurses could not specify the reason for the perceived discrimination. More than 10% of nurses in both groups felt discriminated due to their age, 15% of immigrant nurses due to their origin, ancestry, or skin color, and 14% of non-immigrant nurses due to their family responsibilities and 12% due to their sexual identity.

In the context of qualitative stressors that are typical in the homecare context, non-immigrant nurses (74.7%) felt more burdened by daily taking care of same patients than immigrant nurses (63.8%), but didn’t differ significantly χ² (1) = 3.832, *p* = 0.050. Significantly more immigrant (51.4%) than non-immigrant nurses (35.7%) reported feeling burdened by the confrontation with suffering and dying of patients in the daily care context, χ² (1) = 6.774, *p* = 0.009. More immigrant (65.7%) than non-immigrant nurses (56.1%) felt strained from caring for patients with dementia or other psychiatric disorders, though both groups did not significantly differ χ² (1) = 2.043, *p* = 0.153.

### 3.4. Predictors for Psychosocial Distress

Somatic burden most strongly predicted increased levels of psychosocial distress among immigrant nurses (β = 0.388, *p* = 0, R^2^ = 0.254), as well as non-immigrant nurses (β = 0.389, *p* = 0, R^2^ = 0.394) (Table 4). Age (β = 0.229, *p* = 0.002, R^2^ = 0.092) and full-time employment (β = 0.208, *p* = 0.009, R^2^ = 0.092) also predicted an increase of psychosocial distress among non-immigrant nurses, followed by working overtime (β = 0.163, *p* = 0.008, R^2^ = 0.394) and anxiety (β = 0.199, *p* = 0.008, R^2^ = 0.394).

On the other hand, shift work predicted decreased levels of psychosocial distress among non-immigrant nurses (β = −0.130, *p* = 0.043, R^2^ = 0.394).

Further significant predictors for increasing psychosocial distress among immigrant nurses were influence and freedom at work (β = 0.252, *p* = 0.036, R^2^ = 0.254) and being employed on a fixed-term basis (β = 0.214, *p* = 0.018, R^2^ = 0.254). A functioning relationship with colleagues and supervisors most strongly predicted a decrease in psychosocial distress (β = −0.287, *p* = 0.039, R^2^ = 0.254) among immigrant nurses.

## 4. Discussion

To our knowledge, this was the first study to raise the question of whether immigrant home care nurses are more stressed at work than their non-immigrant colleagues are, and to analyze potential factors, predicting the psychosocial distress of those nurses. We found no significant difference in the psychosocial health-related outcomes among immigrant and non-immigrant homecare nurses. Therefore, immigrant home care nurses in Germany seemed not to be more stressed than non-immigrant nurses. However, the level of stress was high in both groups, and influencing factors on stress differed in both groups to a certain extent.

The comparison of the data with the general population showed that the psychosocial health of this study’s nurses was affected to a high extent. With that said, 25% of this study’s nurses reported depressive symptomatology compared to 7% of the general population [25]. Depressive symptoms were more common among immigrants than non-immigrants in Germany, while less male immigrant nurses showed noticeable depressive symptomatology compared to 21% of their male non-immigrant counterparts under study [25].

Immigrant and non-immigrant nurses reported a lower extent of support by their colleagues and superiors but a higher influence and freedom at work compared to home care nurses, in general, in a similar study [54]. The nurses, in this present study, perceived that their work was being appreciated to a far lower extent than employees, in general, in Germany, while immigrant nurses felt more likely appreciated by their supervisors than do non-immigrant nurses [55].

About one-third of the nurses reported acts of discrimination at work regardless of their ethnic background. Between 2014 and 2015, nearly one-third of the German population reported having been discriminated against, and nearly 50% of those experienced discrimination at their workplace. Age, sexual identity, religion, and ethnic ancestry are the most frequent factors for discrimination [56]. Our data confirmed that age was a leading characteristic of discrimination regardless of ethnic origin, while ethnic origin was the leading discriminatory factor against immigrant nurses.

The extent of the qualitative stressors (BGWmiab) for homecare nurses’ in the present study confirmed the average extent of homecare nurses in Germany. However, in our study, immigrant nurses were more affected. Caring for patients with dementia or other psychiatric illnesses leads to a higher burden compared to nurses, in general, in Germany [57]. Immigrant nurses scored significantly higher than non-immigrant nurses regarding work appreciation by the management. Even though it is evident that experiences of appreciation have a positive effect on job satisfaction and job attitude [58], appreciation did not significantly predict any decrease in psychosocial distress.

More than half of this study’s migrant nurses felt burdened by the confrontation with suffering or dying of patients compared to about a third of non-immigrant nurses. The way how an individual deals with bereavement and grief is influenced by the norms of her or his cultural identity [59]. Cultural dominance and its association with loss and grief through generations can particularly affect migrant and minority people. Migration and acculturation are likely to influence cultural beliefs, world views, and common practices [60]. This may explain why dealing with suffering and dying patients is experienced to a higher extent as burdensome among migrants. However, this burden had a significant effect on psychosocial distress neither among migrant nor on non-immigrant nurses.

Somatic burden most strongly predicted increased levels of psychosocial distress among migrant nurses, as well as non-immigrant nurses. The negative impact of somatic burdens on psychosocial health but also the inverse relationship has been reported in various studies [61,62,63].

Older age was significantly related to psychosocial distress among non-immigrant nurses but not among immigrant nurses, whereas the relationship was negative but not significant. Various studies have reported that the correlation between age and psychosocial stress can either be positive [64,65,66] or negative [67,68,69]. Responsibilities, such as caring for family members or having financial liabilities, like paying off loans, explain higher levels of stress among younger people [64]. On the other hand, prolonged life expectancy can be related to facing more health-related stressors, thus leading to higher levels of perceived stress [70,71].

A functioning relationship with colleagues and supervisors most strongly predicted a decrease in psychosocial distress among immigrant nurses. The longer immigrant nurses were employed at their current employer, the more likely they were to report a positive relationship. Collegial solidarity has a positive effect on organizational commitment, teamwork, working climate, job satisfaction, and inter-hierarchical communication [72], while a good relationship between leaders and staff members promotes mental health and prevents stress [73]. Sharing commonalities, especially for those with a migration background [20,21], could explain this effect among immigrant nurses.

Shift work had a protective effect on the levels of psychosocial distress of non-immigrant nurses. This was inconsistent with most of the previous research, as shift work is connected with obesity, depression, and sleep disturbances [74,75]. However, West and colleagues argued that shift work allows nurses to be more flexible in terms of arranging personal and family duties but also taking care of their own mental hygiene in terms of doing sports and physical activities [76].

Surprisingly, but consistent with previous research in similar disciplines [73,77], a higher extent of influence and freedom at work led to higher levels of psychosocial distress among migrant nurses. Higher levels of qualification or being in a leadership position moderated this relationship. Immigrant nurses with lower qualifications reported a lower extent of influence, while immigrant nurses in leadership positions reported higher levels of influence.

Immigrant nurses felt distressed by fixed-term contractual employment relationships. Even though immigrant and non-immigrant nurses did not differ significantly from each other regarding the proportion of full- or part-time employment, this effect could be explained by societal influences. Immigrant care workers are overrepresented in fixed-term employment relationships [78,79]. The insecurity concerning not knowing how to set up one’s own future of that of their families can have a negative impact on satisfaction and lead to stress [80,81].

The high amount of homecare nurses suffering from somatic burdens, psychosocial distress, depressive symptomatology, and generalized anxiety requires action. Since homecare-nursing takes mostly place “on the move”, behavioral health promotion activities should be designed for the specific circumstances of this setting. There are plenty of lifestyle interventions aiming at improving nurses’ occupational health, which can either solely rely on educational approaches but also being implemented on an organizational level [82]. Most of the following interventions’ efficacy has been proven in randomized controlled trials. Worksite interventions like providing nurses a treadmill at work or Wii system, walking meetings, or health coaching via text messages [83] show a positive effect on nurses’ physical activity. Musculoskeletal complaints that often emerged from psychosocial stressors in the nursing context can be significantly reduced by a combination of coaching interventions, focusing on enabling better strategies for coping and physiotherapy [84]. Interventions that lead to a significant reduction of stress are based on mindfulness/meditation techniques [85], educational approaches like stress and coping behavior techniques [86], light therapy [87], or physical activities like yoga or walking. Depression and anxiety can be significantly reduced by the mindfulness‑based cognitive therapy [88] or education-based interventions [89] or relaxation methods (e.g., feet bath [90], music [90], music-based relaxation [91]).

In our study, nurses reported low levels of appreciation by supervisors and mutual support from colleagues. Toode et al. (2011) explained that these experiences negatively affected nurses’ job motivation and their ability to cope with stress and so they spoke for allowing nurses to exchange with colleagues and supervisors [92]. Additionally, Hunt (2007) stated that making an organization highly performing and its workforce effective, managers and supervisors are advised to value individuals regardless of their ethnic, racial, or cultural background [93].

Reports of discrimination require further actions like detabooing discrimination and all its forms. The nursing personnel must become aware of the severely negative effects of discrimination on the individual’s health, and supervisors have to become competent in evaluating situations as critical and then setting limits to get possible threats under control to the favor of their employees. Corresponding qualification measures are available [94].

We found no significant difference in the extent of psychosocial distress among immigrant and non-immigrant nurses. This could imply that there is no need for special lifestyle interventions for reducing stress and corresponding hazardous threats for each of both groups of nurses. Nevertheless, our data showed and confirmed previous research in that way that both groups differed in stressors and resources associated with distress [20,37]. More efforts are needed on interactional, as well as organizational level, to raise the nurses’, their supervisors’, as well as the clients’ awareness for respecting the individuality of nurses.

The focus of our study was on ambulatory care; however, nursing is not the only industry depending on migrant workers, e.g., agriculture [95]. Therefore, our results might be applicable to these industries as well.

### Strengths and Limitations

Nurses who rate their own health as good might not have participated in this survey that strongly focuses on psychosocial burdens. The low response rate might have led to an overestimation of the symptoms. The group of non-immigrant nurses was overrepresented in our study, and this affected the accuracy of the comparison between both groups. These limitations mentioned it should still be kept in mind that a response rate of 25% is not unusual, and meaning conclusions can still be derived [96]. In future comparative studies, the lower response of immigrant nurses should be considered in the sampling approach by, e.g., oversampling. The documentation of the care process is an integral part of a nurse’s daily work, and the nurse is obliged to document client and care-related issues in the German language. Having said that, this might have led some migrant nurses with limited linguistic competence who were initially interested in participating in revoking their intention, and this could imply an underrepresentation of those nurses. Cross-sectional studies do not allow causal relationships, and thus, the measured outcomes in this study are merely associated with psychosocial distress. In this study, we collected health-related data from nurses working in the homecare sector in the federal state of Hamburg, Germany. While the sample was representative in terms of, e.g., qualification levels, gender, age, and migration background for this specific setting, the results did represent immigrant and non-immigrant nurses neither in Germany, in general, nor nurses in other care-related settings like stationary care. The sociodemographic data showed some significant differences among both groups of nurses under study (e.g., gender, civil status, operated by services, fixed-term contracts). The division of the sample into immigrant and non-immigrant nurses could have led to an underestimation of possible effects among the differences.

This study also has its distinct strengths: A proportional randomized stratified sampling approach had been applied to recruit nursing services in the different districts in Hamburg, which contributed to the representativity of this study’s sample. The applied scales for measuring outcomes related to psychosocial health were widely recognized in psychosomatic healthcare. This allowed an initial assessment of the extent of psychosocial stress and its corresponding factors among immigrant and non-immigrant nurses.

## 5. Conclusions

The extent of psychosocial distress experienced by immigrant and non-immigrant homecare nurses did not significantly differ. However, both groups reported far higher levels of psychosocial distress, depressive symptomatology, generalized anxiety, and somatic burdens than the German general population.

In the homecare sector, nurses were confronted with specific musculoskeletal stressors, such as moving and handling household objects, to gain access to patients in their domestic environment (e.g., non-height adjustable beds), or other typical homecare-related stressors, such as time pressure due to, e.g., traffic jams. Those factors could additionally affect the nurses’ somatic burdensome occupation.

Age was a significant predictor for increased psychosocial distress among non-immigrant nurses. In times of an aging society and nursing workforce, as well as increasing demand for adequate health- and nursing-care but decreasing numbers of qualified nurses, the healthcare system relied on their available and very well-trained nurses. Having said that, nursing managers or supervisors are advised to regularly check their nurses’ psychosocial stress levels to identify any change compared to the last examination. This could be beneficial to the retention of nurses within their home care service providers.

A higher level of influence and freedom at work was related to higher levels of psychosocial distress among immigrant nurses. Immigrant nurses in leadership positions, such as team leader, might benefit from more and continuous internal, as well as external managerial support and training. The negative effect of working overtime on the psychosocial health of non-immigrant nurses confirmed the current blatant lack of nurses.

In times of an increasingly diverse nursing workforce, those responsible in health and nursing care on a macro-, meso-, and micro level should carefully consider the individual explanatory model of psychosocial health of nurses with diverse ethnic and cultural backgrounds.

## Figures and Tables

**Table 1:** Sociodemographic data of the total sample.

	Immigrant Nurses	Non-Immigrant Nurses
**Total**	N = 105	N = 182
**Gender**		
Female *	85.7	73.6
**Age**		
Mean (SD)	43.7 (11.84)	48.11 (11.37)
**Professional status**		
Registered general nurse *	26.7	41.8
Nursing assistant *	10.5	4.4
Geriatric nurse	31.4	35.7
Nursing auxiliary	18.1	13.2
Semi-skilled *	13.3	4.9
**Civil status**		
Single	24.8	22.5
Married *	54.3	35.2
In cohabitation	8.6	16.5
Separated	1.9	4.4
Divorced *	8.6	20.3
Widowed	1.9	1.1
**Operated by services**		
Private-commercial	59.0	59.9
Non-commercial *	12.4	24.2
Don’t know *	28.6	15.9
**Weekly working time**		
Full time (>35 h)	41.9	43.4
Part-time (15–35 h)	49.5	49.5
Part-time (<15 h)	4.8	5.5
Other	3.8	1.6
**Type of contract**		
Permanent contract	80.0	87.4
Fixed-term contract *	20.0	12.6
**First language**		
German *	19.0	98.9
German + other *	23.8	1.1
Other *	49.5	0
More than two other *	7.6	0

Data is % or Mean (SD), * *p* < 0.05.

**Table 2:** Comparison between immigrant and non-immigrant nurses regarding mean scale values.

	Migrant Nurses	Non-Immigrant Nurses	t	df	P
NCCN	5.65 ± 2.17	5.57 ± 2.45	0.284	285	0.777
SSS-8	8.43 ± 4.76	8.20 ± 4.87	0.388	285	0.698
PHQ-9	6.86 ± 5.31	6.67 ± 5.35	0.292	285	0.771
GAD-7	5.83 ± 4.80	5.41 ± 4.66	0.713	285	0.477
COPSOQ					
Influence and freedom at work	49.95 ± 27.70	51.14 ± 21.81	−0.376	179	0.707
Relationship with colleagues and superior	67.53 ± 25.03	62.76 ± 24.04	1.596	285	0.111
Work appreciated by the management	64.67 ± 27.88	57.34 ± 30.55	2,021	285	0.044 *

Comparison between two group means: *t*-test, * *p* < 0.05, Data is Means, NCCN: National Comprehensive Cancer Network Distress Thermometer, SSS-8: Somatic Symptom Scale, PHQ-9: Patient Health Questionnaire, GAD-7: Generalized anxiety disorder, COPSOQ: Copenhagen Psychosocial Questionnaire, df: Degree of Freedom.

**Table 3:** Comparison between immigrant and non-immigrant nurses: the proportion of nurses meeting the cut-off criteria.

	Immigrant Nurses	Non-Immigrant Nurses	χ²	df	Sig.	φ
NCCN	52.4	52.7	0.004	1	0.952	0.952
SSS-8	27.6	24.7	0.291	1	0.589	0.589
PHQ-9	21.9	21.4	0.009	1	0.925	0.925
GAD-7	18.1	15.4	0.357	1	0.550	0.550
ALLBUS	32.4	31.3	0.035	1	0.852	0.852
BGW-miab						
Daily taking care of the same patients	63.8	74.7	3.832	1	0.050	0.050
Confrontation with suffering and dying of patients	51.4	35.7	6.774	1	0.009 **	0.009
Caring for patients with dementia or other psychic diseases	65.7	56.1	2.043	1	0.153	0.153

Comparison between two groups: Chi-Square test, ** *p* < 0.01, Data is %, NCCN: National Comprehensive Cancer Network Distress Thermometer, SSS-8: Somatic Symptom Scale, PHQ-9: Patient Health Questionnaire, GAD-7: Generalized anxiety disorder, ALLBUS: Allgemeine Bevölkerungsumfrage der Sozialwissenschaften, BGW-miab: Berufsgenossenschaft für Gesundheitsdienst und Wohlfahrtspflege - Mitarbeiterbefragung.

**Table 4:** Predictors for psychosocial distress.

	Immigrant Nurses (N = 105)						Non-immigrant Nurses (N = 182)					
	t	β	t	β	t	β	t	β	t	β	t	β
**Step 1**												
Age	−0.339	−0.034	−0.111	−0.011	−0.406	−0.037	2.927	0.214 **	3.108	0.229 **	1.915	0.124
Gender	−0.145	−0.014	−0.579	−0.061	−0.350	−0.033	0.507	0.037	0.331	0.024	1.668	0.103
**Step 2**												
Fulltime			0.247	0.027	0.553	0.055			2.640	0.208 **	2.546	0.166 *
Working overtime			0.068	0.656	0.799	0.073			2.081	0.151 *	2.669	0.163 **
Shift work			1.118	0.113	0.881	0.085			0.220	0.016	−2.035	−0.130 *
Another language than German spoken with clients			0.241	0.024	0.620	0.058			1.827	0.131	1.407	0.084
Fixed−term contract			2.397	0.239 *	2.403	0.214 *			0.209	0.015	0.441	0.027
Registered/Geriatric nurse			−0.403	−0.044	0.266	0.027			1.276	0.097	1.491	0.097
Leadership position			−0.127	−0.014	0.081	0.008			0.058	0.005	−0.249	−0.018
**Step 3**												
Influence and freedom at work (COPSOQ)					2.128	0.252 *					−1.281	−0.092
Relationship with colleagues and superior (COPSOQ)					−2.099	−0.287 *					0.040	0.003
Work appreciated by the management (COPSOQ)					0.177	0.021					−0.384	−0.031
Daily taking care of the same patients (BGWmiab)					0.498	0.055					−0.712	−0.056
Confronted with suffering/dying patients (BGWmiab)					0.259	0.028					−0.170	−0.012
Caring for patients with dementia/psychic diseases (BGWmiab)					0.899	0.097					0.283	0.022
Discrimination (ALLBUS)					0.272	0.029					0.380	0.026
Depressive symptomatology (PHQ−9)					−0.994	−0.154					0.297	0.029
Generalized anxiety (GAD-7)					1.267	0.192					2.005	0.199 *
Somatic symptom burdens (SSS-8)					2.917	0.388 **					4.618	0.389 **
R^2^		−0.018		−0.011		0.254		0.036		0.092		0.394
ΔR^2^		0.001		0.075		0.314 **		0.047 *		0.090 *		0.321 **

Independent variables (step 2) were dummy coded: Yes = 1, No = 0. Dependent variable: psychosocial distress. Hierarchical multiple regression analysis, * *p* < 0.05, ** *p* < 0.01.
